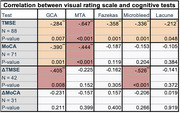# Correlation Between Multiple Visual Rating Scales of Cerebral Small Vessel Lesion and Brain Atrophy with Cognitive Performances in Patients with Amnestic Mild Cognitive Impairment and Mild Dementia in Memory Clinic

**DOI:** 10.1002/alz70856_102388

**Published:** 2025-12-25

**Authors:** Thachamai Smitasiri, Yuttachai Likitjaroen

**Affiliations:** ^1^ Chulalongkorn University, Bangkok, Thailand; ^2^ King Chulalongkorn Memorial Hospital, Bangkok, Thailand; ^3^ Neurocognitive Unit, Division of Neurology, Faculty of Medicine, Chulalongkorn University, Bangkok, Thailand; ^4^ Memory Clinic, King Chulalongkorn Memorial Hospital, The Thai Red Cross Society, Bangkok, Thailand

## Abstract

**Background:**

Cognitive testing and brain MRI studies are essential for diagnosing amnestic mild cognitive impairment(MCI) and mild dementia, suspected from Alzheimer's disease. Identifying simple MRI markers associated with cognitive performance can aid early interventions. This study investigates the relationship between visual rating scales of cerebral small vessel lesions(CSVL) and brain atrophy observed in MRI findings, and cognitive test scores(Thai Mental State Examination;TMSE or Thai translation of the MoCA;MoCA‐Thai) in patients with amnestic MCI and mild dementia.

**Method:**

A cross‐sectional study recruited patients diagnosed with amnestic MCI or mild dementia, suspected from Alzheimer's disease according to clinical criteria of Thai dementia guideline in the Memory Clinic, King Chulalongkorn Memorial Hospital(2020‐2024). In early visit, all participants underwent cognitive testing using either TMSE or MoCA‐Thai as baseline measurement, and brain MRI to exclude other causes including cerebrovascular disease, and to evaluate visual rating scales for CSVL(Fazekas score, microbleeds, and lacunes) and brain atrophy(GCA and MTA scores). Cognitive tests were followed up within 1‐3 years for subset of patients. Spearman's correlation and multiple regression analysis were used to analyze associations between visual rating scales and cognitive test scores.

**Result:**

Among included 100 participants(73 amnestic MCI, 27 mild dementia), results showed moderate negative correlation between MTA scores and baseline cognitive test scores, both TMSE and MoCA‐Thai(R=‐0.647 and ‐0.444, respectively;p<0.001). Weaker correlations were observed in Fazekas score, number of microbleeds and lacunes, correlated to TMSE scores(R=‐0.358, ‐0.336 and ‐0.212, respectively;p<0.05). GCA score and number of microbleeds also showed moderate negative correlations with TMSE score change(ΔTMSE).(R=‐0.405 and ‐0.526, respectively;p<0.01)

Multiple linear regression analysis revealed that MTA score and number of microbleeds were significantly associated with both TMSE and MoCA‐Thai scores(R^2^=0.484 and 0.414, respectively;p<0.001). No visual rating scales demonstrated significant relationship with changes in cognitive test scores over 3‐year period in this study.

**Conclusion:**

This study revealed association between visual rating scales of brain MRI and cognitive performance in amnestic MCI and mild dementia. Hippocampal atrophy appears to have stronger association than CSVL markers. However, further research with larger sample sizes and biomarker confirmation of diagnoses is necessary to elucidate long‐term impact of MRI findings on cognitive decline.